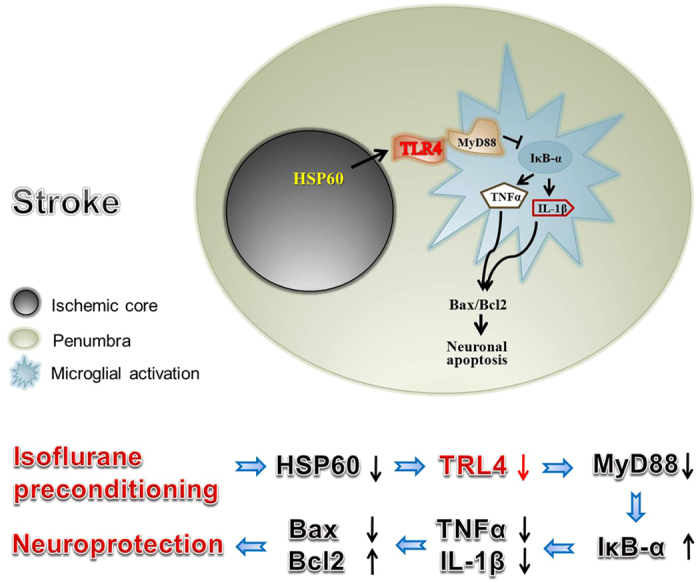# Isoflurane preconditioning provides neuroprotection against stroke by regulating the expression of the TLR4 signalling pathway to alleviate microglial activation

**DOI:** 10.1038/srep11445

**Published:** 2015-06-18

**Authors:** Meiyan Sun, Bin Deng, Xiaoyong Zhao, Changjun Gao, Lu Yang, Hui Zhao, Daihua Yu, Feng Zhang, Lixian Xu, Lei Chen, Xude Sun

**Affiliations:** 1 Department of Anesthesiology, Tangdu Hospital, Fourth Military Medical University, Xi’an 710038, China; 2 Department of Gynaecology and Obstetrics, Nave General Hospital, Beijing, 100059, China; 3 State Key Laboratory of Military Stomatology, Department of Anesthesiology, School of Stomatology, Fourth Military Medical University, Xi’an 710032, China; 4 Department of Anesthesiology, Weifang Medical University, Weifang, Shandong, 261053, China; 5 Department of Medical Administration, Lintong Sanatorium of PLA Lanzhou Military District, Lintong, Xi’an, 710600, China

## Abstract

Excessive microglial activation often contributes to inflammation-mediated neurotoxicity in the ischemic penumbra during the acute stage of ischemic stroke. Toll-like receptor 4 (TLR4) has been reported to induce microglial activation via the NF-κB pathway. Isoflurane preconditioning (IP) can provide neuroprotection and inhibit microglial activation. In this study, we investigated the roles of the TLR4 signalling pathway in IP to exert neuroprotection following ischemic stroke ***in vivo*** and ***in vitro***. The results showed that 2% IP alleviated neurological deficits, reduced the infarct volume, attenuated apoptosis and weakened microglial activation in the ischemic penumbra. Furthermore, IP down-regulated the expression of HSP 60, TLR4 and MyD88 and up-regulated inhibitor of IκB-α expression compared with I/R group ***in vivo***. ***In vitro***, 2% IP and a specific inhibitor of TLR4, CLI-095, down-regulated the expression of TLR4, MyD88, IL-1β, TNF-α and Bax, and up-regulated IκB-α and Bcl-2 expression compared with OGD group. Moreover, IP and CLI-095 attenuated microglial activation-induced neuronal apoptosis, and overexpression of the TLR4 gene reversed the neuroprotective effects of IP. In conclusion, IP provided neuroprotection by regulating TLR4 expression directly, alleviating microglial activation and neuroinflammation. Thus, inhibiting the activation of microglial activation via TLR4 may be a new avenue for stroke treatment.

Ischemia/reperfusion (I/R)-induced brain injury is implicated in the pathophysiology of stroke and results in an initial area of neuronal death known as the core, surrounded by an area vulnerable to further damage known as the penumbra^1^. Furthermore, neuronal apoptosis and necrosis in the penumbra can be viewed as primary causes of aggravated cerebral injury and functional impairment. Additionally, neuronal apoptosis can be exacerbated by the excessive formation of inflammatory reactions after an ischemic stroke, which has been demonstrated to be the one cause contributing to cerebral I/R injury^2^. Although several studies have been devoted to developing methods to reduce inflammatory reactions during ischemic cerebral injury, effective methods have not yet been established.

Inflammatory processes are involved in a broad range of pathologies and diseases of the central nervous system (CNS) including stroke, brain trauma and brain infection^3^. Previous studies have found that acute inflammatory cascade reactions that cause overactivation of microglia and the release of inflammatory substances can aggravate neuronal injury in the cerebral penumbra^4^. Microglia are macrophage-like cells that are considered the major immune cells in the brain^4^,^5^. During the response to various endogenous or exogenous stimuli, microglia can be activated to produce inflammatory cytokines such as IL-1, TNF and inducible nitric oxide (iNOS), which can induce neuronal apoptosis and aggravate brain injury^6^,^7^. Therefore, reducing microglial overactivation in the penumbra could be beneficial and might provide neuroprotection against cerebral ischemia.

Isoflurane is commonly used in the clinic and is a relatively safe volatile anesthetic. Many studies have shown that isoflurane preconditioning (IP) can induce ischemic tolerance and exert neuroprotection against cerebral I/R injury^8^,^9^,^10^,^11^. Zuo found that rats that were pretreated with 2% isoflurane could reduce neuronal apoptosis and regulate the expression of anti-apoptotic protein Bcl-2 after stroke *in vivo*^12^ and attenuate microglial activation *in vitro*^13^. However, despite the important role of IP in inhibiting microglial activation, little information is available regarding how IP reduces microglial activation and provides neuroprotection during the process of the ischemic stroke.

TLRs are key mediators of innate immunity that respond to injury-induced endogenous ligands from necrotic cells such as HSPs and diverse microbial products^14^. In particular, Toll-like receptor 4 (TLR4) is expressed primarily in microglia^15^,^16^,^17^. We and other studies have reported that TLR4 can induce microglial activation and cytokine production in brain injury, trauma and neurodegenerative diseases^18^,^19^. Moreover, Hara *et al.* reported that TLR4 knockout (KO) mice had significantly smaller infarct volumes and better neurobehavioural recovery at 24 h after I/R injury compared with wild-type mice. Furthermore, these authors found that attenuated the nuclear factor kappa-light-chain-enhancer of activated B cells (NF-κB) activation in TLR4 KO mice can provide neuroprotection during acute focal cerebral I/R injury^20^. Another study also showed that TLR4 expression in activated microglia mediated neuroinflammation via an NF-κB signalling pathway in response to hypoxia and that TLR4 inhibition can attenuate TNF-α and IL-1 expression against hypoxia^21^. Hence, microglial TLR4 may be as a potential therapeutic target to inhibit microglial activation for ischemic stroke treatment.

Therefore, in the current study, we demonstrated that TLR4 is necessary for microglial activation triggered by I/R insult, which mediated neuroinflammation via an NF-κB signalling pathway and resulted in neuronal apoptosis. Moreover, we investigated the putative roles and mechanisms by which IP could exert neuroprotection by regulating the expression of the TLR4 signalling pathway to alleviate microglial activation after cerebral I/R injury *in vivo* and *in vitro*.

## Materials and Methods

### Animals

Adult male Sprague-Dawley (SD) rats (280–320 g), SD rat embryos (E17.5) and SD rat pups (1–2 d old) were provided by the Experimental Animal Centre of the Fourth Military Medical University (Xi’an, China). SD rats used in the *in vivo* studies were divided randomly into 3 groups: sham operation (Sham), cerebral I/R (I/R), and IP followed by cerebral I/R (IP + I/R). Preconditioned animals were exposed to 2% isoflurane for 30 min at 24 h before ischemia. Focal cerebral ischemia was induced by middle cerebral artery occlusion (MCAO). Rats in the Sham group underwent similar surgical procedures without MCAO. Then, parietal brain tissues were harvested for the next step in the study. SD rat embryos (E17.5) were used for neuron/astrocyte and neuron cultures, and SD rat pups (1–2 d old) were used for microglial cell cultures. All animal experiments were conducted in accordance with the National Institutes of Health Guide for the Care and Use of Laboratory Animals (NIH publications number 80-23, revised in 1996). The experimental protocol was approved by the Ethics Committee for Animal Experimentation and was conducted according to the Guidelines for Animal Experimentation of the Fourth Military Medical University.

### Isoflurane preconditioning

Twenty-four hours before MCAO, rats were anesthetized with isoflurane via a facemask followed by endotracheal intubation and mechanical ventilation. Anesthesia was maintained with 2% isoflurane in a mixture of air and oxygen (fraction of inspired oxygen, 0.25–0.3) for 30 min. The gas pressure was monitored continuously. Respiration was controlled by a ventilator to maintain normal end-tidal O_2_ and CO_2_ concentrations. The inhaled and exhaled gases were monitored with a Datex infrared analyser (Capnomac, Helsinki, Finland). After IP, the rats were extubated and returned to their cages when adequate ventilation was resumed. The Sham and I/R groups were treated for the same duration with only the mixture of air and oxygen via endotracheal intubation as in the IP + I/R group.

*In vitro*, microglia were placed in an airtight chamber gassed with 95% air/5% CO_2_ through an isoflurane vaporiser set at 2% for 30 min at 37 °C. The isoflurane concentrations in the gases from the outlet of the chambers were monitored with a Datex infrared analyser (Capnomac, Helsinki, Finland) and reached the target concentration at approximately 3 min after the onset of gassing. At the end of the incubation period, the isoflurane concentrations in the gases from the outlet of the chamber were confirmed to be at the target concentration. For the groups without IP, the microglia were treated identically but without isoflurane. After exposure, the cells were allowed to recover at 37 °C in a humidified incubator under 95% air/5% CO_2_^13^.

### Transient focal cerebral ischemia

The MCAO-induced model of transient focal cerebral ischemia in rats was performed as described previously^22^,^23^. Briefly, the rats were anesthetized with 2% chloral hydrate during surgical preparation at 24 h after isoflurane exposure. The right middle cerebral artery of the rats was occluded by the insertion of a 3-0 nylon monofilament suture (Ethicon Nylon Suture, Ethicon Inc., Sukagawa, Japan). The regional cerebral blood flow (rCBF) was monitored using a disposable microtip fibre optic probe (diameter, 0.5 mm) connected to a computerised laser Doppler primary unit (PeriFlux 5000, Perimed AB, Sweden) through a master probe. Rats retaining >20% of baseline perfusion during ischemia were excluded. Reperfusion was accomplished by withdrawing the suture after 90 min of ischemia, followed by suturing the surgical wounds. The animals’ temperatures were monitored and maintained at 37.0–37.5 °C by surface heating or cooling during surgery until the rats recovered from anesthesia. After recovery from anesthesia, the rats were placed back in their cages and housed with free access to food and water.

### Evaluation of neurological deficit scores (NDSs) and infarct volume

NDSs were evaluated at 24 h after reperfusion based on an eight-point scale by an investigator who was blinded to the animal grouping. The rats were scored as follows: 0, no apparent deficits; 1, failure to extend left forepaw fully; 2, decreased grip of the left forelimb; 3, spontaneous movement in all directions, contralateral circling only if pulled by the tail; 4, circling or walking to the left; 5, walking only if stimulated; 6, unresponsiveness to stimulation and with depressed level of consciousness; 7, dead^24^.

Then, the infarct volume was detected as described previously^25^. Briefly, the rats were decapitated, and 2-mm-thick coronal sections throughout the brain were prepared, stained with 2% 2,3,5-triphenyltetrazolium chloride (TTC) (Sigma, USA) for 30 min at 37 °C, and then fixed in 4% paraformaldehyde for 24 h. The stained slices were imaged using a digital camera. The infarction area in each slice was measured using the software Image-Pro Plus 6.0 (Media Cybernetics, USA). The total infarct volume in the brain was the sum of the infarct volume of each brain slice. Infarct volume (%) = (contralateral hemisphere volume-ipsilateral normal hemisphere volume)/contralateral hemisphere volume × 100%.

### Terminal-deoxynucleotidyl transferase-mediated dUTP-biotin nick end labelling (TUNEL) staining

To determine whether IP reduced apoptosis, TUNEL staining was performed *in vivo* and *in vitro* using an *In Situ* Cell Death Detection Kit (Roche Diagnostics, Mannheim, Germany) as described previously and according to the manufacturer’s instructions^26^. In brief, sections at bregma +1.5 mm in the ipsilateral hemisphere (penumbral cerebral cortex)^12^ and neurons on the plates were processed for TUNEL staining. The sections or neurons in every group were fixed in 4% (v/v) ice-cold paraformaldehyde for 1 h, washed in PBS (0.1 M, pH 7.4) for 5 min, treated with 0.3% (v/v) H_2_O_2_ for 10 min, rinsed in PBS for 5 min, and incubated in a TUNEL reaction mixture for 1 h at 37 °C (after which the neurons were stained with DAPI for 5 min at room temperature). Images were obtained using a microscope (BX60, Olympus). Five sections from each rat were selected randomly, and neurons from five separate fields on each coverslip were counted. The average number of positive cells was counted for each individual rat or coverslip by 2 investigators who were blinded to the treatment.

### Immunofluorescence (IF) staining

The extent of microglial activation and the expression of TLR4 were evaluated by IF staining, and all sections for staining were obtained from the ischemic penumbral tissue at the level of bregma +1.5 mm in the ipsilateral hemisphere. Before Iba1 labelling, the tissues underwent antigen retrieval by incubating the sections for 10 min in a solution of Tris-buffered saline (TBS) containing 20 μg/ml proteinase K, followed by a 10-min incubation in distilled water containing 3% H_2_O_2_. The sections were pre-incubated in PBS containing 0.1% Triton X-100 and 2% normal goat serum for 24 h, and then transferred into the same solution containing rabbit polyclonal anti-Iba1 antibody (1:500; Wako Chemicals USA, Inc., Richmond, VA, USA) for 48 h at 4 °C. Then, the sections were incubated with Alexa Fluor 594-conjugated donkey anti-rabbit IgG (1:500; Molecular Probes, Rockford, IL, USA) secondary antibody for 1 h at 37 °C. Finally, DAPI (1 ng/μl; Roche) was added to stain the nuclei. For TLR4 labelling, the sections were pre-incubated in PBS containing 0.1% Triton and 2% normal goat serum for 0.5 h and then transferred into the same solution containing mouse monoclonal antibody against the microglial marker CD11b (1:200, Abcam, Cambridge, MA, USA) and mouse monoclonal anti-TLR4 (1:50; Abcam, USA) for 24 h at 4 °C. Then, the sections were incubated with FITC-conjugated goat anti-mouse IgG (1:200; Vector, Burlingame, CA, USA) secondary antibody. Finally, five sections from each rat were selected randomly and analysed using a Leica DMLB fluorescence microscope (Leica Microsystems, Wetzlar, GmbH, Germany). Images were captured using an Olympus FluoView FV10i confocal laser scanning microscope (Olympus Corp., Shinjuku, Tokyo, Japan). All stained sections were examined and analysed by an investigator who was blinded to the treatment.

### Western blot analysis

Western blot analysis was used to detect the expression of heat shock protein 60 (HSP60), TLR4, myeloid differentiation factor 88 (MyD88) MyD88 and nuclear factor κB-α (IκB-α) in the cerebral ischemic penumbra at 24 h after reperfusion as described previously^27^. Briefly, rats were anaesthetised deeply and decapitated, and their brains were removed quickly. Total protein from each ischemic penumbra was acquired using an extraction kit (KeyGen, Nanjing, China) on ice. The following primary antibodies were used: mouse anti-HSP60 (1:5000, Abcam), mouse anti-TLR4 (1:100; Abcam), rabbit anti-MyD88 (1:200, Abcam), mouse anti-IκB-α (1:500, Novus Biologicals) and mouse anti-actin (1:1000, Abcam). Western blot analysis was also used to detect the expression of HSP60, TLR4, MyD88, IκB-α, Bax and Bcl2 in the *in vitro* study, as described previously^26^. The total protein concentration of the cells was analysed using a BCA kit (KeyGen, Nanjing, China). The blots were probed with mouse anti-HSP60 (1:5000, Abcam), mouse anti-TLR4 (1:100; Abcam), mouse anti-MyD88 (1: 200, Abcam), mouse anti-IκB-α (1:500, Novus Biologicals) mouse anti-Bcl2 (1:200, Aobo), or mouse anti-Bax (1:200, Aobo). Secondary horseradish peroxidase (HRP)-conjugated goat anti-rabbit antibody or goat anti-mouse antibody (Pierce Biotechnology Inc; 1:5000 dilution) was used. The signals were detected using an ECL kit (Pierce) according to the manufacturer’s instructions. The changes in relative protein expression were represented as the ratio of the integrated optical density of the protein bands to that of β-actin. The experiments were performed independently in triplicate.

### Primary cell culture

#### A. Neuron/astrocyte culture

Neuron/astrocyte cultures were prepared as described previously^28^. Briefly, the cortices of SD rat embryos (E 17.5) were dissected out and triturated in 0.25% trypsin for 15 min at 37 °C. The triturated tissue was added to DMEM with 20% FBS to neutralise the trypsin, and the solution was vortexed and then centrifuged at 1,000 rpm for 5 min. A portion of the cells were resuspended in serum-free culture Neurobasal culture medium (Gibco, Invitrogen Corp., USA) that contained 2% B27 supplement, 1% glutamine and 1% penicillin/streptomycin (Sigma-Aldrich Corp., USA) at 37 °C in a humidified incubator with 95% air and 5% CO_2_. The cultured cortical cells were seeded onto poly-L-ornithine-treated (Sigma, St. Louis, MO) coverslips (Bellco Glass, Vineland, NJ) at 3 × 10^4^ cells/well and grown for 7–10 d to increase the proportion of mature neurons. Every 4 d, 50% of the medium was replaced with fresh Neurobasal/B27 medium without antioxidants. After 7 d, the culture purity was determined by staining the mature neurons with microtubule-associated protein 2 (MAP2) (1:100; Abcam, USA) and the astrocytes with GFAP (1:500; Abcam, USA); approximately 70% of the cells were neurons, and 30% were astrocytes (data not shown).

#### B. Neuronal culture

Primary neurons were cultured as described previously^26^. Briefly, cerebral cortices were harvested from SD rat embryos (E 17.5). Then, the tissue was dissociated by trituration in DMEM containing 0.25% trypsin and 0.02% EDTA (Hyclone) followed by incubation for 10 min at 37 °C with agitation. DMEM with 10% FBS was added to neutralise the trypsin, and the solution was vortexed and then centrifuged at 2,000 rpm for 5 min. A portion of the cells were grown in Neurobasal medium (Gibco, Invitrogen Corp., USA) supplemented with 2% B27, 1% glutamine and 1% penicillin/streptomycin (Sigma-Aldrich Corp., USA) at 37 °C in a humidified incubator with 95% air and 5% CO_2_. At 5 days post culture, the purity of the neurons and astrocyte was determined by immunocytochemistry using mouse monoclonal anti-βIII-tubulin (1:250; Millipore, Temecula, CA, USA) and rabbit monoclonal anti-GFAP (1:500; Abcam, USA) antibodies. At 7 days post culture, the purity of the neurons indicated that 95% of the cells in cultures were positive for βIII-tubulin (data not shown).

#### C. Microglial culture

Primary microglia were cultured as described previously^13^,^28^. Briefly, cerebral cortices were harvested from SD rat pups (1–2 d old). The dissociated brain tissue was minced in DMEM containing 0.25% trypsin and 0.02% EDTA (Hyclone) and then incubated for 10 min at 37 °C with agitation. DMEM with 10% FBS was added to neutralise the trypsin, and the solution was vortexed and then centrifuged at 1,000 rpm for 10 min. The pelleted cells were resuspended and seeded in 75 cm^2^ flasks (Corning) containing DMEM supplemented with 10% FBS for microglia culture. The media was changed every 3 days. At 12 days post culture, the flasks were shaken at 150 rpm for 40 min at 37 °C to release the loosely adherent microglia population from the adherent astrocyte monolayer. The cells were collected and microglia cultures were maintained in DMEM with 10% FBS and were ready for use in subsequent experiments 3 days later. Iba1 staining was performed to confirm that microglia cultures were >90 to 95% pure (data not shown).

### OGD model

To test the neuroprotective effects of IP against ischemic injury *in vitro*, the previously described OGD model was used in this study^27^,^28^. First, the primary neurons/astrocytes were cultured on coverslips in 24-well plates in Neurobasal media without glucose (Invitrogen, Carlsbad, CA, USA) in a humidified incubator filled with an anoxic gas mixture (5% CO_2_ and 95% N_2_) at 37 °C for 1, 2, 3, and 4 h, separately. Two h of OGD caused ~40% of the neurons to become TUNEL positive, and this duration was used for all subsequent experiments (data not shown).

### The ***in vitro*** penumbra model

The *in vitro* penumbra model was performed as described previously^28^.

- Neuron/astrocyte (~70:30%) cultures were subjected to 2 h OGD by growing them on the lower well of a Transwell chamber (Corning) to simulate the stroke core.
- These OGD-stressed neuron/astrocyte (~70:30%) cultures (OGD-sn) were co-cultured with microglia in the porous upper insert of the Transwell chamber for 24 h, allowing molecules released from the OGD-sn cultures to activate the nearby microglia (95% air, 5% CO_2_, 37 °C). The 3-μm-diameter pores allow molecules to diffuse between the OGD-sn and microglia, which are separated by ~1 mm, without permitting cell-cell contact. This stage was designed to represent the expanding penumbra.
- To model the developing penumbra, the microglia were washed with media and allowed to interact with healthy neurons (95% pure) on the lower chamber for 48 h. This approach was used to examine whether microglia were activated by OGD-sn and whether the activated microglia produced neurotoxic signals that subsequently damaged the healthy neurons.

### Enzyme-linked immunosorbent assay (ELISA)

The levels of interleukin-1beta (IL-1β) and tumour necrosis factor-alpha (TNF-α) in the neuronal supernatant were measured using a Quantikine ELISA kit according to the manufacturer’s protocol. Briefly, the groups were designed as described above. At 48 h of co-culture with healthy neurons (95% pure), 50 μl of the supernatant was loaded into a microplate that was pre-coated with monoclonal antibodies specific for IL-1β (1:100, anti-rabbit polyclonal, Millipore, USA) and TNF-α (1:100; anti-rabbit polyclonal, Millipore, USA). After washing, an enzyme-linked polyclonal antibody specific for rat IL-1β and TNF-α was added. Substrate solutions were added to the wells following washing. The intensity of the enzyme reaction was measured using an ELISA reader (Epoch^TM^, BioTek, USA) at 450 nm.

### TLR4 DNA transfection

TLR4 DNA was transfected into the microglia to overexpress TLR4. TLR4 DNA (pEX-3) and control DNA (pEX-3) were transfected into cells using Lipofectamine 2000 (DNA:Lipofectamine 2000 = 1 μg:2.5 μl). The microglia were used for experiments at 24 h after transfection^29^.

### Neuron viability assay

At 48 h of co-culture with healthy neurons (95% pure), neuron viability was assessed using the MTT assay as described previously^30^. Briefly, MTT was added to the cells after treatment. In metabolically active cells, the yellow tetrazolium MTT salt is cleaved into purple formazan crystals, which can be solubilised, and then the absorbance is measured using a multi-plate reader at 490 nm.

### ***In vitro*** experimental protocols

- To investigate whether OGD-sn cultures released HSP60, the neurons/astrocytes (~70:30%) were divided into two groups: OGD (neurons/astrocytes that received OGD) and Normal (normal neurons/astrocytes). After 2 h of OGD, the neurons/astrocytes were scraped off for western blot analysis of HSP60 protein expression.
- Next, we investigated the effects of IP and the TLR4 inhibitor CLI-095 (C15H17ClFNO4S; 3950 Sorrento Valley Blvd. Suite 100, San Diego, CA 92121 - USA) on penumbral microglia and on neurons. In the Normal group, microglia were cultured without any treatments. In the OGD group, microglia were co-cultured with OGD-sn for 24 h, washed and allowed to interact with healthy neurons (95% pure) for 48 h. In the IP + OGD group, microglia were pretreated with 2% isoflurane for 30 min at 24 h before the OGD-sn stimulus. Then, the microglia were co-cultured with OGD-sn for 24 h, washed and allowed to interact with healthy neurons (95% pure) for 48 h. In the CLI-095 + OGD group, microglia were incubated with 1 μmol CLI-095 for 1 h before OGD-sn stimulus^31^ and then co-cultured with OGD-sn for 24 h. Subsequently, the microglia were washed and allowed to interact with healthy neurons (95% pure) for 48 h. After the OGD-sn stimulus, the microglia were scraped off for western blot analysis of TLR4, MyD88 and IκB-α protein expression. At 48 h of co-culture with healthy neurons (95% pure), the treated microglial supernatants were used to measure the levels of IL-1β and TNF-α by ELISA, and the cells were divided into two assays to determine the level of apoptosis of the treated neurons, The two assays were western blot analysis of Bcl-2 and Bax protein expression and TUNEL staining.
- To examine the hypothesis that IP exerts neuroprotection by directly regulating TLR4 expression, microglia were divided into five groups: Normal, OGD, IP + OGD, IP + OGD + TLR4 DNA (IP + OGD + TLR4 DNA overexpression), IP + OGD + Control (Con) DNA. Microglia in the Normal, OGD, and IP + OGD group underwent similar procedures as the groups in protocol B. After 24 h of transfection and simultaneous stimulus with OGD-sn, the microglia were scraped off for western blot analysis of TLR4, MyD88 and IκB-α protein expression. At 48 h of co-culture with healthy neurons (95% pure), the viability of the treated neurons was assessed using the MTT assay as described previously.

### Statistical analysis

SPSS software (version 11.1; SPSS, Chicago, IL) was used to conduct the statistical analyses. All values, except for neurological scores, were presented as the means ± standard error of the mean (SEM) and were analysed by one-factor analysis of variance (ANOVA). The neurological scores were expressed as the median (range) and were analysed using the Kruskal-Wallis test followed by the Mann-Whitney U-test with the Bonferroni correction. Values of P < 0.05 were considered statistically significant.

## Results

### IP (2%) alleviated neurological deficits and reduced infarct volume after stroke

**Figure 1:**
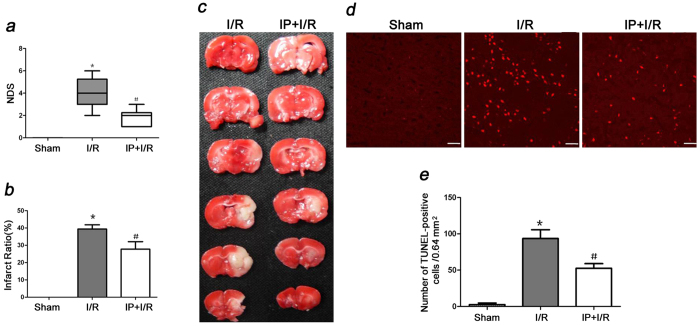
Preconditioning with 2% isoflurane alleviated neurological deficits, reduced infarct volumes, and attenuated apoptosis in the cerebral ischemic penumbra after stroke (n = 6). (**a**) The NDSs were recorded in each animal 24 h after reperfusion. The NDSs of the IP + I/R group were significantly better than were those of the I/R group 24 h after reperfusion. The results are expressed as the median (range). ^*^P < 0.05 vs. Sham group, ^#^P < 0.05 vs. I/R group. (**b**) Representative brain infarct size according to TTC staining at 24 h after reperfusion. (**c**) Statistical analysis of the infarct sizes of each group. The results are shown as the means ± SEM. ^*^P < 0.05 vs. Sham group, ^#^P < 0.05 vs. I/R group. (**d**) Representative TUNEL staining (red) at 24 h after reperfusion. Scale bars = 100 μm. (**e**) Statistical analyses of the numbers of TUNEL-positive cells. The results are shown as the means ± SEM. ^*^P < 0.05 vs. Sham group, ^#^P < 0.05 vs. I/R group.

At 24 h after reperfusion, the NDSs in the IP + I/R group were higher than were those in the Sham group (P < 0.05). However, the NDSs in the IP + I/R group were lower than were those in the I/R group (P < 0.05) (Fig. 1a).

Additionally, the infarct ratio in the I/R group increased compared to that of the Sham group (P < 0.05) at 24 h after reperfusion. In contrast, the infarct ratio in the IP + I/R group decreased compared to that of the I/R group (P < 0.05) (Fig. 1b,c).

### IP (2%) attenuated apoptosis in the cerebral ischemic penumbra

The number of TUNEL-positive cells in the cerebral ischemic penumbra in the I/R group was greater than that in the Sham group (P < 0.05). Nevertheless, the number of TUNEL-positive cells in the cerebral ischemic penumbra in the IP + I/R group was less than that in the I/R group (P < 0.05) (Fig. 1d,e).

### IP (2%) significantly weakened microglial activation in the cerebral ischemic penumbra after reperfusion

**Figure 2:**
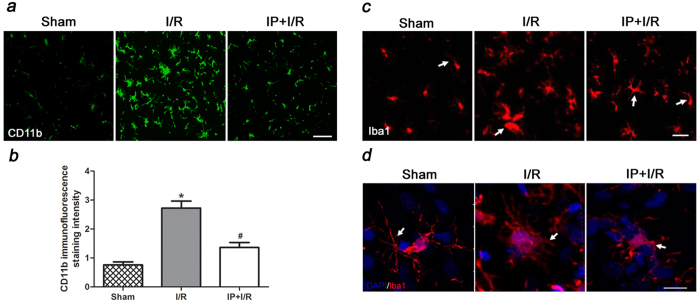
Preconditioning with 2% isoflurane significantly weakened microglial activation in the cerebral ischemic penumbra (n = 6). (**a**) Immunofluorescence staining for CD11b-positive microglia (green) in the cerebral ischemic penumbra at 24 h after I/R. Scale bars = 50 μm. (**b**) Statistical analyses of the CD11b IF staining intensity of activated microglia in the cerebral ischemic penumbra at 24 h after reperfusion. The results are shown as the means ± SEM. ^*^P < 0.05 vs. Sham group, ^#^P < 0.05 vs. I/R group. (**c**) Immunofluorescence staining for Iba1-positive microglia (red) in the cerebral ischemic penumbra at 24 h after I/R. The white arrows indicate processes. Scale bars = 50 μm. (**d**) Immunofluorescence staining and three-dimensional reconstruction of microglia in the cerebral ischemic penumbra at 24 h after I/R. The three-dimensional reconstruction of a single microglia indicates that the activated microglia exhibit a transformation in cellular morphology. The microglia are double-labelled with Iba1 (red) and DAPI (blue). The white arrows indicate processes. Scale bars = 20 μm.

The microglia were identified in sections from normal brain or the cerebral ischemic penumbra using CD11b and Iba1 labelling. The CD11b-positive microglia were hypertrophic and exhibited characteristic bushy cellular ramifications, which were distributed extensively in the cerebral ischemic penumbra (Fig. 2a). We also analysed CD11b IF staining intensity of the activated microglia in the cerebral ischemic penumbra at 24 h after reperfusion (Fig. 2b). The IF intensity of the CD11b-positive microglia increased significantly in the I/R group compared to the Sham group (P < 0.05) and decreased significantly in the IP + I/R group compared to the I/R group (P < 0.05). Iba 1 staining and the three-dimensional reconstruction of a single microglia showed that the microglia were at rest and exhibited a ramified cell morphology with massive thin processes extending away from their soma in the Sham group. In contrast, at 24 h after reperfusion, the microglia in the cerebral ischemic penumbra were activated, the thin processes were drawn back into their soma, and their cell bodies were larger than were those in the Sham group. However, the microglia soma were smaller and the processes were longer in the IP + I/R group than in the I/R group, which indicated that the extent of microglial activation was weakened by 2% IP (Fig. 2c,d).

### The effect of IP on the expression of the HSP60/TLR4/MyD88/IκB-α signalling pathway in the cerebral ischemic penumbra after stroke

**Figure 3:**
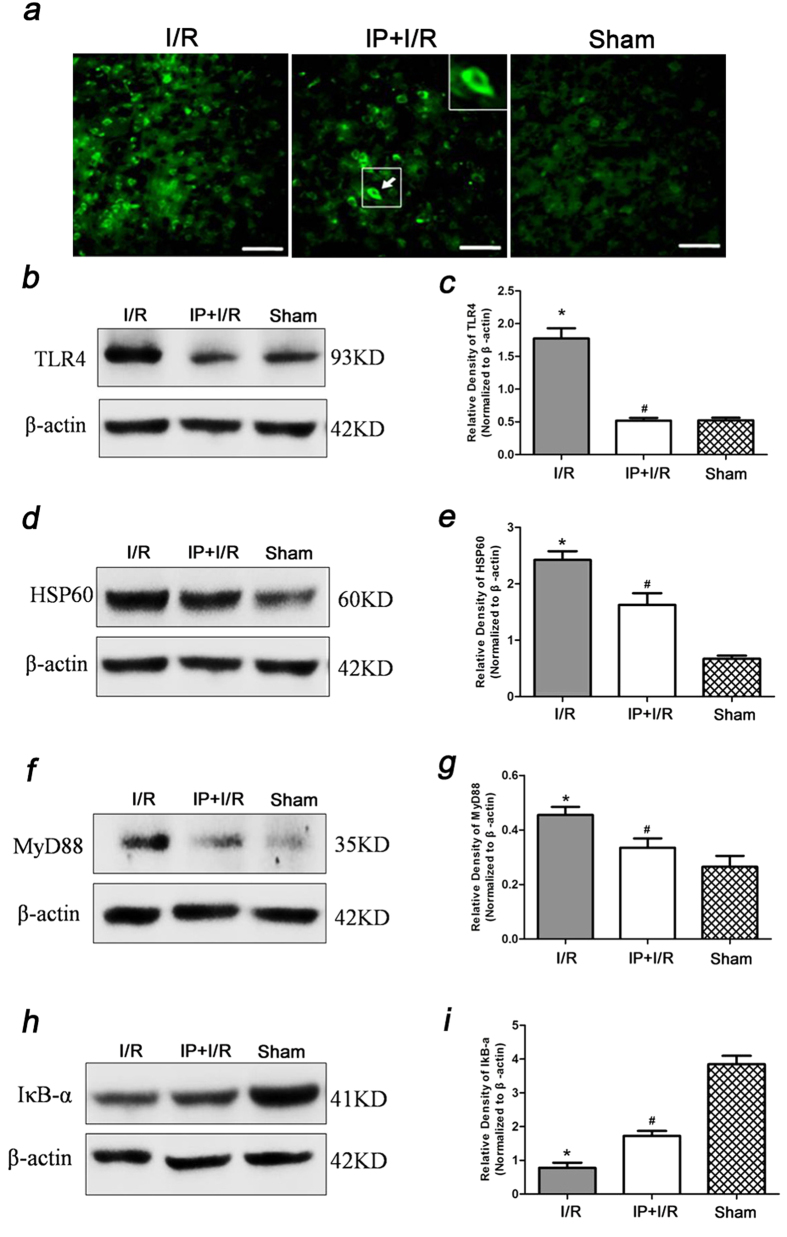
The effect of 2% IP on HSP60/TLR4/MyD88/IκB signalling pathway expression in the cerebral ischemic penumbra (n = 6). (**a**) Images showing the distributions and expression of TLR4 (green) immunoreactive cells in the cerebral ischemic penumbra at 24 h after reperfusion for every group. Scale bars = 50 μm. (**b**–**i**) Effects of 2% IP on HSP60, TLR4, MyD88 and IκB-α protein levels in the cerebral ischemic penumbra at 24 h after reperfusion. The gels/blots were cropped for better showing figures. The data are presented as the means ± SEM. ^*^P < 0.05 vs. Sham group, ^#^P < 0.05 vs. I/R group.

At 24 h after reperfusion, the IF showed that TLR4 was primarily expressed in the cytoplasmic membrane in the cerebral ischemic penumbra. Both the IF and western blot showed that TLR4 expression was higher in the I/R group compared to the Sham group, while TLR4 expression was lower in the IP + I/R group compared to the I/R group in the cerebral ischemic penumbra (Fig. 3a–c).

We also detected HSP60, MyD88 and IκB-α protein expression in the cerebral ischemic penumbra. The results showed that HSP60 and MyD88 protein expression was up-regulated, while IκB-α protein expression was evidently down-regulated in the IP + I/R group compared to the Sham group (P < 0.05). However, HSP60 and MyD88 protein expression was down-regulated, and IκB-α was up-regulated in the IP + I/R group compared to the I/R group (P < 0.05) (Fig. 3d–i).

### HSP60 protein expression in the OGD-sn ***in vitro***

**Figure 4:**
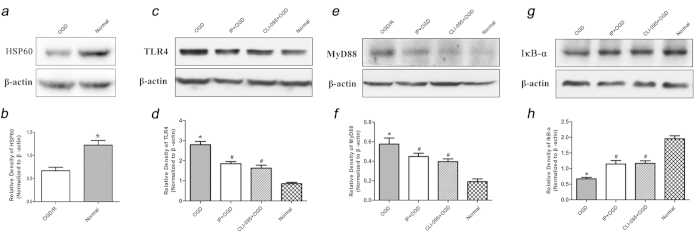
HSP60 protein levels of OGD-sn cultures and the effects of 2% IP and CLI-095 treatment on TLR4/MyD88/IκB signalling pathway expression in the penumbra model ***in vitro***. (**a**,**b**) HSP60 protein levels in the OGD-sn cultures after OGD injury (n = 6). The data are presented as the means ± SEM. ^*^P < 0.05 vs. Normal group. (**c**–**h**) Effects of 2% IP and CLI-095 treatment on TLR4, MyD88 and IκB-α protein levels in treated microglia after the OGD-sn stimulus (n = 6). The gels/blots were cropped for better showing figures. The data are presented as the means ± SEM. ^*^P < 0.05 vs. Normal group, ^#^P < 0.05 vs. OGD group.

To determine the mechanism of TLR4 activation after the microglia were exposed to OGD-sn cultures, we first investigated HSP60 protein expression in the OGD-sn cultures. The result showed that HSP60 protein expression was obviously up-regulated in the OGD group compared to the Normal group (P < 0.05) (Fig. 4a,b). HSP60 is one of the endogenous ligands of TLR4. Therefore, this result suggested that the increase in HSP60 protein expression may be one reason for the up-regulation of TLR4 after the microglia were exposed to the OGD-sn stimulus.

### The effect of IP and CLI-095 on TLR4/MyD88/IκB-α expression in treated microglia from the ***in vitro*** penumbra model

After the OGD-sn stimulus, microglial TLR4 and MyD88 expression in the OGD group was significantly higher than that in the Normal group (P < 0.05). In contrast, TLR4 and MyD88 expression in the IP + OGD group or CLI-095 + OGD group was significantly lower than that in the OGD group (P < 0.05) (Fig. 4c–f). Additionally, this result showed that IκB-α expression in the OGD group was significantly lower than that in the Normal group (P < 0.05). However, IκB-α expression increased following IP or CLI-095 treatment (P < 0.05) (Fig. 4g,h). These results suggested that IP could weaken the microglia reaction by inhibiting TLR4 expression and downstream signal transduction.

### IL-1β and TNF-α expression in treated microglia was attenuated by IP and CLI-095 ***in vitro***

**Figure 5:**
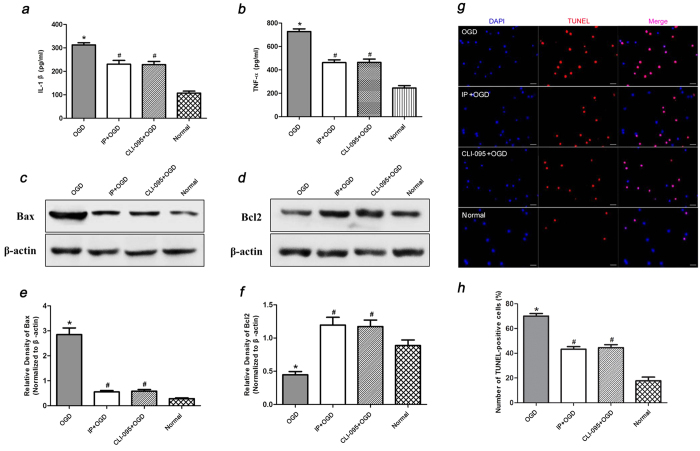
IP attenuated neuronal apoptosis by influencing microglial activation via an immune inflammatory reaction ***in vitro***. At 48 h of co-culture with healthy neurons (95% pure), the effects of IP and CLI-095 treatment on microglial activation-induced (**a**) IL-1β and (**b**)TNF-α release in neuronal supernatant were measured by ELISA (n = 6). Additionally, (**c**,**e**) Bcl-2 and (**d**,**f**) Bax expression were measured by western blot analysis (n = 6) (The gels/blots were cropped for better showing figures), and (**g**,**h**) the numbers of TUNEL-positive neurons were counted (n = 6). The neuronal nuclei were labelled with DAPI (blue) and TUNEL (red). The data are presented as the means ± SEM. ^*^P < 0.05 vs. Normal group, ^#^P < 0.05 vs. OGD group.

IL-1β and TNF-α expression in the supernatant was detected by ELISA at 48 h of co-culture with healthy neurons (95% pure). IL-1β and TNF-α expression was higher in the OGD group compared to that in the Normal group (P < 0.05). However, IL-1β and TNF-α expression decreased in the IP + OGD or CLI-095 + OGD group compared to that in the OGD group (P < 0.05) (Fig. 5a,b). These results indicated that OGD could induce microglial activation and that the cultures released a large amount of IL-1β and TNF-α, which are inflammatory mediators. Moreover, IP and CLI-095 could attenuate this type of inflammatory reaction.

### IP and CLI-095 attenuated microglial activation-induced neuronal apoptosis ***in vitro***

Neuronal apoptosis was analysed by determining the expression of the apoptosis-related proteins Bax and Bcl-2 and by TUNEL staining at 48 h of co-culture with healthy neurons (95% pure). Bax protein expression increased in the OGD group compared to that in the Normal group (P < 0.05). In contrast, isoflurane exposure or CLI-095 treatment significantly decreased Bax expression compared to OGD (P < 0.05) (Fig. 5c,e). Additionally, the amount of Bcl-2 protein was lower in the OGD group compared to that in the Normal group (P < 0.05). In contrast, isoflurane exposure or CLI-095 treatment significantly increased Bcl-2 expression compared to OGD (P < 0.05) (Fig. 5d,f). Furthermore, the number of TUNEL-positive cells decreased by IP and CLI-095 treatment compared to OGD (P < 0.05) (Fig. 5g,h). These results implied that microglial activation may lead to neuronal apoptosis and that both IP and CLI-095 treatment could alleviate neuronal apoptosis by regulating the expression of apoptosis-related proteins.

### TLR4 DNA overexpression reversed the neuroprotective effects of IP

**Figure 6:**
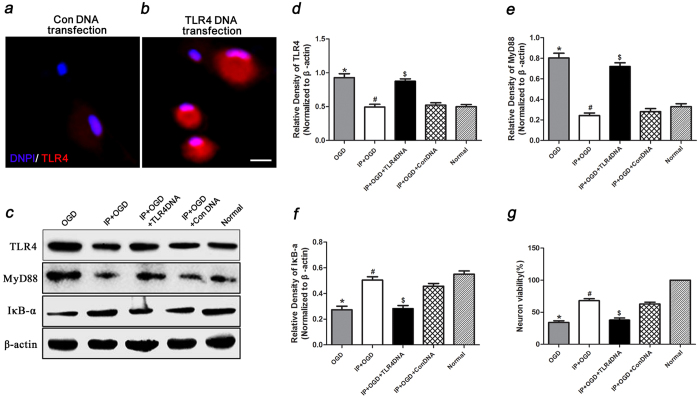
TLR4 DNA transfection reversed the effects of IP on TLR4, MyD88 or IκB-α expression in microglia and aggravated neuron injury. (**a**,**b**) TLR4 expression (red) was detected by IF staining of microglia that received IP treatment and the OGD-sn stimulus at 24 h after TLR4 DNA and Con DNA transfection; DAPI staining is in blue. Scale bar = 10 μm. (**c**) Effects of TLR4 DNA transfection on TLR4, MyD88 and IκB-α protein levels after 24 h of transfection. (**d**–**f**) Statistical analysis of the western blot assay (n = 6) (The gels/blots were cropped for better showing figures). The data are presented as the means ± SEM. ^*^P < 0.05 vs. Normal group, ^#^P < 0.05 vs. OGD group. ^$^P < 0.05 vs. IP + OGD group or IP + OGD + Con DNA group. (**g**) The effects of IP and TLR4 DNA transfection on neuron viability at 24 h after transfection (n = 6). ^*^P < 0.05 vs. Normal group, ^#^P < 0.05 vs. OGD group. ^$^P < 0.05 vs. IP + OGD group or IP + OGD + Con DNA group.

After 24 h of TLR4 DNA and Con DNA transfection, the expression of TLR4 in microglia was detected by IF staining. The staining intensity in the TLR4 DNA transfection group was higher than the Con DNA transfection group (Fig. 6a,b). TLR4, MyD88 and IκB-α expression was also tested. The TLR4 and MyD88 protein levels in the OGD group or IP + OGD + TLR4 DNA group were higher than those in the IP + OGD group or IP + OGD + Con DNA group (P < 0.05). In contrast, IκB-α expression was lower in the OGD group or IP + OGD + TLR4 DNA group than that in the IP + OGD group or IP + OGD + Con DNA group (P < 0.05) (Fig. 6c–f). Moreover, the MTT assay showed that neuron viability was lower in the OGD group or IP + OGD + TLR4 DNA group than in the IP + OGD group or IP + OGD + Con DNA group (P < 0.05) (Fig. 6g). These findings suggested that TLR4 overexpression following TLR4 DNA transfection abrogated the effects of IP on TLR4, MyD88 and IκB-α expression in microglia and on neuron survival. Therefore, this study suggested that IP exerts neuroprotection by inhibiting the function of TLR4 and its downstream signalling molecules, which can evoke inflammatory reactions and regulate the expression of apoptosis-related proteins (Fig. 7).

## Discussion

Inflammation appears to play a key role in the pathogenesis of ischemic stroke. Experimentally^32^,^33^ and clinically^34^,^35^, the brain responds to ischemic injury with an acute and prolonged inflammatory process. Multiple lines of evidence suggest that microglia, the resident macrophages of the brain, are activated during this acute process^36^,^37^. Microglia play a dual role in the central nervous system, as they have both neurotoxic and neuroprotective effects^38^,^39^. Excessive activation of microglia often contributes to inflammation-mediated neurotoxicity through the release of proinflammatory mediators, including IL-1β and TNF-α^6^,^7^,^39^. Early studies have shown that microglia orchestrate inflammation in the penumbra^5^. In our study, we also found that microglia in the penumbra were activated at 24 h after I/R *in vivo*. Moreover, the activated microglia released a large amount of IL-1β and TNF-α, inflammatory mediators that induced neuronal apoptosis *in vitro*. These findings are consistent with the previous studies described above.

Although evidence that microglia orchestrate inflammation in the penumbra exists, the actions of these cells are not fully understood. The TLRs, so-called because of their homology to the *Drosophila* Toll receptor, were first characterised in mammals by their ability to recognise pathogen-associated molecular patterns (PAMPs). Recently, TLRs have been shown to act as sentinels of tissue damage and mediators of inflammatory responses to aseptic tissue injury, in addition to their roles in pathogen detection and defence^14^. TLR4 is expressed primarily in microglia in the CNS^15^,^17^, which can be activated by either exogenous (such as LPS)^40^ or endogenous ligands (such as HSP60)^41^ to induce downstream signals (MyD88-dependent and MyD88-independent signals) that lead to cytokine and chemokine production and thereby initiate inflammatory responses. HSP60 has been described previously as an endogenous ligand of TLR4^42^,^43^. Previous studies have shown that CNS injury leads to the release of HSP60, which subsequently activates innate immunity in a TLR4- and MyD88-dependent manner *in vitro*^43^. Previous studies have shown that TLR4-MyD88 is a key signalling pathway in ischemic penumbral microglial activation^44^,^45^ and that the TLR4-MyD88 signalling pathway is activated by HSP60 after I/R injury and may be involved in the mechanism of hypoxia by up-regulating NF-κB and TNF-α^46^. NF-κB is a downstream factor of the TLR4-MyD88 signalling pathway^47^ and is present in an inactive state in the cytoplasm when bound to the inhibitory IκB subunit^48^. NF-κB is activated by a variety of external stimuli that phosphorylate IκBs via IκB kinase (IKK). When IκBs are phosphorylated, NF-κB is released and migrates to the nucleus, triggering the transcription of genes involved in the inflammatory response^49^. Therefore, we detected the degree of NF-κB activation via the reduction of IκB in our current study. Furthermore, TLR4 can promote the production of major inflammatory mediators such as TNF-α and IL-1β, which have been implicated in neuronal apoptosis and death^47^. Another report has shown that TNF-α and IL-1β can be activated by NF-κB activation after ischemic injury^50^ and that NF-κB pathway activation is responsible for TLR4-induced target gene expression after hypoxic treatment in microglia^51^.

Combining all of the theories above, we hypothesised that HSP60 was released from the ischemic core and activated TLR4-MyD88 on the resting microglia, which activated the transcription factor NF-κB. Subsequently, the activated microglia released TNF-α and IL-1β, which killed the penumbral neurons by an apoptotic pathway. To demonstrate the above hypothesis accurately, we used two types of model *in vivo* and *in vitro* experiments in this study. For the *in vivo* experiment, we selected the transient MCAO model. This model can imitate acute stroke, which causes an irreversibly damaged ischemic core and salvageable surrounding tissue known as the penumbra. The penumbra was the pharmacological target for acute ischemic stroke treatment^52^. For the *in vitro* experiment, we selected a more appropriate model of the stroke penumbra. To recapitulate inflammatory triggers in the core, microglia were exposed to OGD-sn cultures. To model the developing penumbra, the microglia were washed and allowed to interact with healthy neurons^28^. In the present study, we found that HSP60/TLR4/MyD88/NF-κB expression was up-regulated in the penumbra after I/R *in vivo*. Moreover, HSP60 in OGD-sn cultures and TLR4/MyD88/NF-κB/TNF-α/IL-1β in microglia presented the same trends, along with additional TUNEL-positive neurons after the OGD-sn stimulus in the *in vitro* experiment. This result was similar to Kaushal and Schlichter’s study^28^, where OGD-sn cultures evoked NF-κB activation in microglia and resulted in neurotoxic activity. Most importantly, CLI-095, an inhibitor of TLR4, down-regulated the expression of microglial TLR4/MyD88/NF-κB/TNF-α/IL-β and decreased the number of TUNEL-positive neurons in the penumbra *in vitro*. Therefore, we confirmed that the penumbral microglial TLR4-MyD88 signalling pathway participated in the mechanisms of penumbra injury after I/R; however, whether HSP60 that was released from the core led to signal transduction remains unclear and requires further study.

Recently, much attention has been given to therapeutic strategies for neuroprotection that are aimed at inhibiting neurotoxic microglial activation. Isoflurane, a commonly used volatile anesthetic, has been shown to have neuroprotective effects in many studies^8^,^10^,^11^. Our current study also showed that IP reduced brain infarct volumes, alleviated neurological deficits, and decreased the density of TUNEL-positive cells in the penumbral cerebral cortex after I/R, consistent with previous studies^12^. In our study, we selected 2% isoflurane for 30 min at 24 h before brain ischemia based on previous studies^53^. In brief, 1) One minimum alveolar concentration (the concentration to inhibit 50% of subjects from responding to surgical stimuli) of isoflurane for adult rats is approximately 1.4%^54^,^55^, and 2% isoflurane is approximately equivalent to 1.4 minimum alveolar concentrations, which is often used in clinical practice. 2) A duration of 15 to 30 min is required for the IP to be maximally protective^56^. 3) We administered the preconditioning stimulus 24 h before brain ischemia because isoflurane is metabolised poorly and washed out of the body quickly^57^. Thus, we postulated that all of the isoflurane inhaled during preconditioning had been thoroughly cleared from the rats before brain ischemia.

Some evidence has indicated that 2% IP can reduce mouse microglial activation *in vitro*^13^. Consistently, our study also found that 2% IP weakened microglial activation and affected TLR4-MyD88 expression in the penumbra after I/R *in vivo*. How isoflurane weakens microglial activation and whether TLR4-MyD88 is involved in this process remain unclear. Our results showed that IP and CLI-095 had similar effects on the expression of microglial TLR4/MyD88/NF-κB/TNF-α/IL-1β and on the reduction of apoptosis in penumbral neurons. Our findings suggested that the TLR4 signalling pathway is directly related to IP, attenuating microglial activation in I/R-induced ischemic brain injury. Furthermore, the present study also found that the well-known anti-apoptotic protein Bcl-2 is implicated in the IP-induced improvement of neurological outcomes after stroke, as reported in previous studies^58^. One of the important mechanisms by which Bcl-2 provides protection is the inhibition of cytochrome c release from the mitochondria, therefore, preventing the activation of apoptotic pathways^59^,^60^. Additionally, our study also suggested that IP and CLI-095 both up-regulated neuronal Bcl-2 expression *in vitro*, which is consistent with our TUNEL results and with previous studies. We also tested the expression of Bax, another apoptotic protein. Although our result differed from that of Zuo’s study^12^,^58^, our result suggested that IP and CLI-095 could alter Bax expression in the *in vitro* penumbra model. The reason may be related to the use of the OGD and microglial activation model in the *in vitro* experiments. This model can activate microglia and release TNF-α, IL-1β or other inflammatory mediators, which exert inflammation-mediated neurotoxicity and result in altered Bax expression and neuronal apoptosis.

We also overexpressed TLR4 by DNA transfection in cells to further elucidate the inhibitory effects of IP on the TLR4 signalling pathway. The results showed that TLR4 overexpression following TLR4 DNA transfection abrogated the protective effects of IP on microglial TLR4, MyD88 and NF-κB protein expression and on neuron viability after treated microglia were co-cultured. These results further demonstrated that IP is actually protective because it directly regulates TLR4 expression.

In summary, we confirmed a theory that damaged neurons in the ischemic core release HSP60, as an endogenous ligand of TLR4, which may activate the microglial TLR4-MyD88 signalling pathway and then activate microglia in the ischemic penumbra. Next, the activated microglia propagate the damage to healthy neurons in the penumbra via the inflammatory molecules TNF-α or IL-1β. However, IP provides neuroprotection by regulating the TLR4 signalling pathway directly, alleviating microglial activation in the ischemic penumbra (as shown in Fig. 7). Our findings also imply that inhibiting microglial activation via TLR4 may be a new avenue of therapy for stroke treatment. Of course, further investigations are needed to elucidate the complete mechanisms of these effects.

## Additional Information

**How to cite this article**: Sun, M. *et al.* Isoflurane preconditioning provides neuroprotection against stroke by regulating the expression of the TLR4 signalling pathway to alleviate microglial activation. *Sci. Rep.* **5**, 11445; doi: 10.1038/srep11445 (2015).

## Supplementary Material

Supplementary Information

## Figures and Tables

**Figure 7:**