# Supplementary material for: Isoflurane preconditioning provides neuroprotection against stroke by regulating the expression of the TLR4 signalling pathway to alleviate microglial activation
Source: Sci Rep. 2015 Jun 18;5:11445. doi: 10.1038/srep11445 (PMC4471883; doi:10.1038/srep11445)
Supplement: Supplementary Information [file srep11445-s1.doc]

**Supplementary information**

**Isoflurane preconditioning provides neuroprotection against stroke by** **regulating the expression of the TLR4 signalling pathway to alleviate microglial activation**

Meiyan Sun^a†^, Bin Deng^a,c†^, Xiaoyong Zhao^a,d†^, Changjun Gao^a^, Lu Yang^a^, Hui Zhao^a^, Daihua Yu^a^, Feng Zhang^e^, Lixian Xu^c^, Lei Chen^b^*, Xude Sun^a^*

**Figure 1**


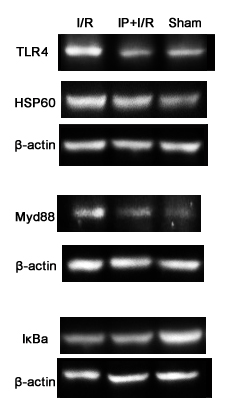


Figure 1. Effects of 2% IP on HSP60, TLR4, MyD88 and IκB-α protein levels in the cerebral ischemic penumbra at 24 h after reperfusion tested by western blot analysis. The full-length gels/blots were cropped for better showing figures in the manuscript.

**Figure 2**


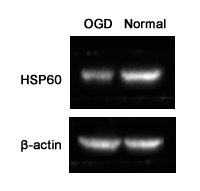


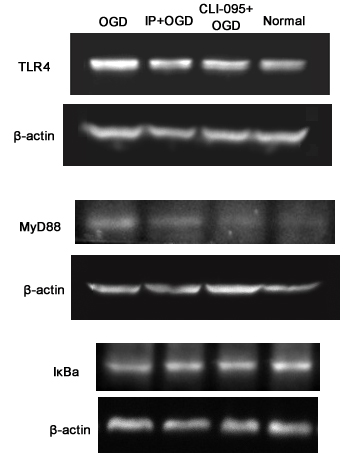


Figure 2. HSP60 protein levels in the OGD-sn cultures after OGD injury measured by western blot analysis. Effects of 2% IP and CLI-095 treatment on TLR4, MyD88 and IκB-α protein levels in treated microglia after the OGD-sn stimulus measured by western blot analysis. The full-length gels/blots were cropped for better showing figures in the manuscript.

**Figure 3**


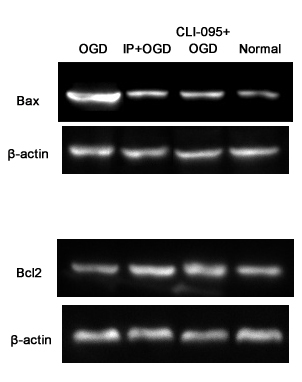


Figure 3 Bcl-2 and Bax expression were measured by western blot analysis. The full-length gels/blots were cropped for better showing figures in the manuscript.

**Figure 4**


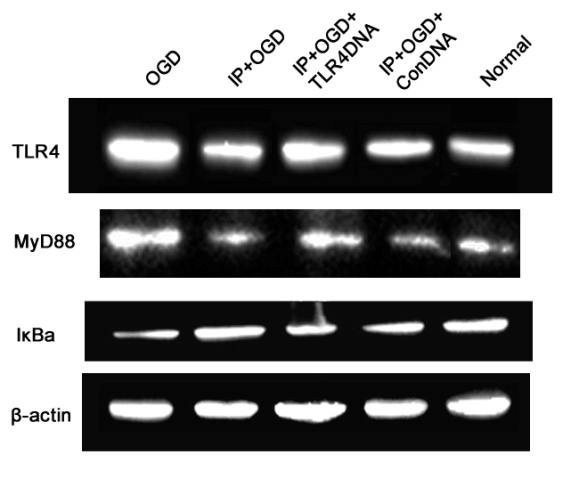


Figure 4. Effects of TLR4 DNA transfection on TLR4, MyD88 and IκB-α protein levels after 24 h after TLR4 DNA and Con DNA transfection tested by western blot assay. The full-length gels/blots were cropped for better showing figures in the manuscript.
